# Mucosa: Key Interactions Determining Sexual Transmission of the HIV Infection

**DOI:** 10.3389/fimmu.2019.00144

**Published:** 2019-02-06

**Authors:** Sandra M. Gonzalez, Wbeimar Aguilar-Jimenez, Ruey-Chyi Su, Maria T. Rugeles

**Affiliations:** ^1^Grupo Inmunovirología, Facultad de Medicina, Universidad de Antioquia, Medellín, Colombia; ^2^National HIV and Retrovirology Laboratory, JC Wilt Infectious Diseases Research Centre, Public Health Agency of Canada, Winnipeg, MB, Canada; ^3^Department of Medical Microbiology and Infectious Diseases, University of Manitoba, Winnipeg, MB, Canada

**Keywords:** HIV exposure, HIV infection, mucosal transmission, mucosal immunity, HIV trans-infection, vaginal microbiota, dendritic cells

## Abstract

In the context of HIV sexual transmission at the genital mucosa, initial interactions between the virus and the mucosal immunity determine the outcome of the exposure. Hence, these interactions have been deeply explored in attempts to undercover potential targets for developing preventative strategies. The knowledge gained has led to propose a hypothetical model for mucosal HIV transmission. Subsequent research studies on this topic further revealed new mechanisms and identified new host-HIV interactions. This review aims at integrating these findings to inform better and update the current model of HIV transmission. At the earliest stage of virus exposure, the epithelial integrity and the presence of antiviral factors are critical in preventing viral entry to the submucosa. However, the virus has been shown to enter to the submucosa in the presence of physical abrasion or via epithelial transmigration using paracellular passage or transcytosis mechanisms. The efficiency of these processes is greater with cell-associated viral inoculums and can be influenced by the presence of viral and immune factors, and by the structure of the exposed epithelium. Once the virus reaches the submucosa, dendritic cells and fibroblasts, as recently described, have been shown *in vitro* of being capable of facilitating the transfer of viral particles to susceptible cells, leading to viral dissemination, most likely in a trans-infection manner. The presence of activated CD4^+^ T cells in submucosa increases the probability of infection, where the predominant microbiota could be implicated through the modulation of an inflammatory microenvironment. Other factors such as genital fluids and hormones could also play an essential role in HIV transmission. Here, we review the most recent evidence described for mucosal HIV-transmission contributing with the understanding of this phenomenon.

## Introduction

HIV infection remains one of the most critical health problems worldwide, with close to 37 million people infected in 2017 (1). Despite the low rate of sexual transmission, sexual intercourse still accounts for the majority of global infections, making it the main transmission route (2). In the context of HIV sexual transmission at genital mucosa, initial interactions between the virus and mucosal immune effector mechanisms determine the outcome of exposure. The first barrier for viral entry into the submucosa is the epithelium, whose structural conformation is variable along the genital mucosa with important implications regarding viral transmission (3). Except for the endocervix and anal mucosa, composed by a monolayer of columnar epithelium, most of the genital mucosa consists of several layers of keratin-containing cells, known as stratified squamous epithelium that presents a major physical barrier to viral entry (4). Accordingly, the anorectal epithelium exhibits the highest probability of HIV-transmission (0.3–5%) (5, 6) in comparison to female (0.05–0.5%) (5) and male genital epithelium (0.04–0.14%) (5, 7), followed by the oral mucosa (0.01%) that is the least susceptible epithelium (5, 6).

The virus, either cell-free or cell associated, can penetrate the epithelium through micro-lacerations that occur during sexual intercourse, via paracellular passage after HIV-triggered epithelial disruption, or by transcytosis across epithelial cells (8). These processes can be modulated by the presence of several factors, including variations in the expression of viral receptor and co-receptors on immune cells, local pro-inflammatory environment, mucosal antiviral factors, HIV-cell interactions, hormonal levels, the composition of the commensal microbiota, and pathogenic co-infections (9–11). Indeed, the frequency of penile-vaginal transmission of HIV was reported to be as high as 10% and penile-anal transmission as high as 33% depending on those risk cofactors (7, 12).

After the virus passes the epithelial barrier, dendritic cells (DC), Langerhans cells (LC), and the recently described fibroblasts at the submucosal space have been shown *in vitro*, and *ex vivo* to transfer viral particles to susceptible CD4^+^ T cells, mainly in the trans-infection manner (13–15); this greatly facilitates the spread of viral infection (16, 17). The establishment of productive HIV-infection however, is highly influenced by the activation status of CD4^+^ T cells, their response profile (preferential infection of Th17), and their location at the genital tract.

Although frequent exposure to HIV often results in infection, some individuals remain uninfected, despite repeated exposure. They are known as HIV-exposed seronegative individuals (HESNs) and have been identified and characterized in various cohorts in attempts to identify mechanisms underlying the resistant phenotype. Some of the well described mechanisms include: (i) the lack of expression of the viral co-receptor CCR5 (18); (ii) increased production of the chemokines MIP-1α/β, RANTES or SDF-1 (19); (iii) apoptosis of target cells (20); (iv) high expression of anti-HIV factors like SLPI, Defensins, Cathelicidin, TRIM5α, APOBEC-3G, SAMHD-1, Serpina1, and Elafin (21, 22); (v) reduced IRF-1 expression (23, 24); (vi) increased activity of natural killer (NK) (25, 26), and dendritic cells (DC) (27); (vii) the presence of neutralizing IgA antibodies (26, 28); and (viii) an effective and polyfunctional response of HIV-1-specific CD4^+^ and CD8^+^ T cells (29, 30). Most of these resistance mechanisms have been observed at the mucosa of HESNs, highlighting the importance of the initial interactions between the virus and the mucosal immune system in predicting the eradication or establishment, and dissemination of the infection. In this regard, many studies focusing on defining the most critical steps during HIV mucosal exposure and transmission have contributed to a hypothetical model in attempts to discover potential targets for the development of preventive strategies (31). The intense research on this topic has brought many novel aspects of HIV-transmission into the light, including novel interactions and factors implicated, allowing to deepen the current knowledge. For example, delineating viral strategies in inducing the loss of tight junctions, uncovering host factors that favor viral transcytosis through epithelial cells, defining cells subsets that participate in viral transfer to susceptible target cells at genital mucosa, and realizing the role(s) of hormones, microbiota, and genital fluids in affecting the cellular susceptibility of immune cells to viral infection. This review has grouped these new findings with the previously defined model, providing a holistic model of HIV transmission at genital mucosa.

### HIV and Early Mucosal Interactions

#### Source of Transmitted HIV: Cell-Free or Cell-Associated Virus?

The first crucial aspect to consider in early mucosal HIV transmission is the source of the transmitted virus. It is widely accepted that HIV is present in the female genital secretions (32) and semen from HIV-infected men (33), with the amount of virus influencing the rate of transmission (34, 35). However, a less defined aspect is the source of the transmitted virus; whether it comes from cell-free virions or infected cells. Both, cell-free virions as well as HIV-infected cells (T lymphocytes and macrophages) have been found in genital secretions (36) and can interact with epithelial tissue (37), transmitting infection during sexual intercourse as demonstrated *in vivo* in animal models (38, 39). However, HIV transmission by cell-associated HIV seems to be more efficient than by cell-free virions in male, female and anorectal mucosa (39–45). One likely explanation might be related with the close contact established between infected and susceptible target cells, where the presence of membrane protrusions and cell engulfment processes during the infectious or virological synapses, facilitate cell-to-cell viral transmission (40). Indeed, it has been described that the interaction between HIV infected cell and the epithelial target cells induces the budding of viral particles toward the epithelium, leading to productive infection of epithelial cells *in vitro* (46); nonetheless, the susceptibility of these cells remains controversial (47–49).

#### Role of Genital Fluids and Antiviral Factors in HIV Transmission

During the initial interaction of the virus with the epithelium, the presence of antiviral peptides like defensins provide the first line of defense against the virus, targeting viral glycoproteins and inhibiting cell binding and fusion (50); then, virions can be trapped by mucus (51) and physically expelled out of the genital tract. In addition, in the oral mucosa most cell-free viruses entering the epithelium are intracellularly inactivated by beta-defensins 2 (HBD2) and 3 (HBD3), and SLPI (52). However, the activity of the antiviral peptides appears to vary at different epithelial sites, in part, due to differential expression of antiviral genes such as HBD2, HBD3, Elafin, SAMHD1, Serpina1, APOBEC3G, Trim5α, and RNase 7 (21), being highly expressed in oral epithelial cells, compared to cervical epithelial cells (52).

Specifically, cervico-vaginal fluids (CVF) are composed of vaginal transudate, mucus, antimicrobial factors, chemokines, and cytokines, including defensins, SLPI, Elafin, RANTES, and CCL2 that have been associated with protection against HIV infection (53). Nonetheless, the antiviral activity of CVF varies depending on the viral strain (54), their location at the female reproductive tract (FRT) (53) and the pH (55). Indeed, an efficient trapping of the HIV by mucus was only observed in presence of an acidic pH (55).

The role of semen in HIV transmission at the FRT is still controversial. On one side, semen may be the culprit of transporting the virus to the upper FRT. Beyond its role in viral transportation, semen can also induce changes at the FRT, increasing the vaginal pH, that could alter the ability of cervical mucus to trap the HIV (55) and also modulating the immune response to promote favorable conditions for conception and pregnancy (56). Such an effect impact the transmission of HIV (56). Indeed, semen can induce expression of various inflammatory genes and the release of pro-inflammatory cytokines and chemokines, increasing the number of susceptible targets at the FRT (56). It has also been described that the amyloid fibrils in semen, and the heparan sulfate on spermatozoids can bind the virus, enhancing the infection of CD4^+^ T cells or DC, respectively (56, 57). By the other hand, it has been described that semen can block viral entry into mucosa (42), that could be related to the presence of several inhibitory factors like cationic polypeptides and reactive oxygen species with anti-HIV activity in semen (58, 59). In addition, semen can also prevent, *in vitro* viral transfer, by blocking viral attachment to DC-SIGN receptor in DC (60). Therefore, the role of semen in female HIV-1 transmission remains an important topic of research.

#### Epithelial Structure at Mucosal Level

Beyond the presence of antiviral peptides, the composition and structure of the exposed epithelium are also crucial in terms of susceptibility to viral entry. The anorectal epithelium exhibits the highest probability of HIV-transmission (0.3–5%) in comparison to the female (0.05–0.5%) and male genital epithelium (0.04–0.14%) (5, 7), followed by the oral mucosa (0.01%) that is the least susceptible epithelium (5–7). The oral mucosa is a highly stratified epithelium with tight junctions observed between the more superficial monolayers, which obstruct access of viral particles to the submucosa. These tight junctions are formed by dimerization between trans-membrane proteins, such as occludin and claudins, in association with the cytoplasmic protein zonula occludens (ZO-1) that together maintain the polarized structure of the epithelium (31).

The adult human foreskin is a stratified epithelium consisting of two different facets: the outer foreskin, being the external surface highly keratinized, and the inner foreskin being a mucosal “wet” epithelium with a lower degree of keratinization. The inner epithelium has a higher frequency of HIV target cells such as CD4^+^ T lymphocytes, Langerhans, macrophages and DCs (61–64); indeed, most of the available evidence indicates that the inner layer is more susceptible to HIV infection than the outer foreskin (43, 64, 65). Remarkably, in circumcised men, mucosal foreskin epithelium is removed leaving a dry keratinized epithelial surface which is more resistant to HIV infection as demonstrated *in vitro* and *in vivo* (66, 67).

The penile urethra is also potentially important for HIV transmission, in both non-circumcised and circumcised men. The urethra is covered by a stratified squamous epithelium and the mucosal urethral epithelium contains immune cells such as macrophages and T cells but not Langerhans although HIV targets first macrophages (61, 62, 65).

Although ectocervical and vaginal mucosae also consist of multi-stratified epithelium, their tight junctions are only found in the deepest monolayers of cells (31). Several immune cells such as intraepithelial CD4^+^ T cells, DC, and macrophages, located at ectocervical and vaginal mucosae, have been shown to be more targeted by incoming viral particles (3), a fact that could partially contribute to explain the higher rate of genital, vs. oral transmission (31).

Nonetheless, compared to the endocervical mucosa, ectocervical and vaginal mucosae may still provide a better protective barrier (56), as at the endocervix, the epithelium is formed by a single layer of columnar cells, allowing the virus to achieve closer proximity to intraepithelial and submucosal target cells (10). Moreover, transcytosis of virions has been shown to occur through polarized columnar epithelial cells (68–70); finally, although still controversial, viral transcytosis might also occur through squamous epithelial cells (3, 71). In addition, the transformation zone, a squamous-columnar junction where stratified ectocervical epithelium converges with the endocervical columnar epithelium, has also shown to be particularly susceptible to HIV infection due to its location and vulnerable structure (4). These reports suggest that, even at the same mucosa, different regions provide a diverse interacting environment with the virus resulting in a different degree of HIV susceptibility. In the case of the rectal mucosa, the simple columnar structure of the epithelium increases the susceptibility to HIV infection, compared to the previously described mucosa (31). Also, the presence of M cells at gut mucosa could potentiate the infection (72); however, there is not sufficient evidence supporting the presence of M cells at the rectal epithelium (73).

### Epithelial Translocation of HIV

Furthermore, the virus can pass across the mucosal epithelium by two mechanisms: (i) *paracellular passage* and (ii) *transcytosis*; it is proposed that the choice of one or the other may rely on the type and the intrinsic characteristic of the epithelium (3).

#### HIV-Triggered Epithelial Disruption Favors HIV Paracellular Passage

*Paracellular passage* could occur virtually in all mucosal types. It results from a loss or disruption of tight junctions triggered by X4 and R5 tropic laboratory strains, clinical isolates and by HIV soluble proteins, causing the formation of gaps at the epithelial monolayers; then, through these gaps the virus may reach the submucosa (31, 74, 75). The epithelial disruption has also been observed *in vivo* by immunohistological analysis of a macaque SIV model after 2.5 days post-SIV exposure, despite low viral loads in peripheral blood and gut mucosa without CD4^+^ T cell loss (74).

Specifically, binding of the HIV glycoprotein 120 (gp120) to either the co-receptors CCR5, CXCR4, or galactosylceramide (GalCer), expressed on epithelial cells was shown to result in a reduction of the expression of occludin, claudins, and ZO-1. This process was associated with an increase in Ca^2+^ levels and the activation of MAPK and PI3K pathways leading to destabilization of ZO-1 and Claudins/Occludin interaction, disruption of tight junctions, internalization of surface proteins, and epithelial monolayers disruption (75–78).

In addition, host antiviral mediators can also weaken the mucosal epithelium. High levels of pro-inflammatory cytokines such as TNF-α can induce the activation of Myosin light-chain kinase (MLCK) that phosphorylates MLC leading to reorganization of actin-cytoskeleton, destabilization of tight-junction protein ZO-1, and internalization of other tight-junction proteins claudins and occluding (31). Recognition of viral particles by the toll-like receptors (TLRs) on epithelial cells has been shown to induce TNF-α production via activating NF-kB-mediated pathways, resulting in internalization of tight junction proteins (79). Other described mechanisms for mediating the disruption of tight junctions involve the activation of the apoptosis pathway in which the increase of Caspases allows cleavage and degradation of claudins, occludin and ZO-1 proteins in epithelial cells (80).

Although *Paracellular passage* has been observed *in vitro* in proportions as low as 0.1% (74), their impact in HIV transmissions *in vivo* is unknown.

#### HIV Translocation Across Epithelial Cells by Transcytosis

The second mechanism is *transcytosis* that takes advantage of the vesicular/endosomal transport machinery of the cells. *Transcytosis* has been described mainly for the columnar epithelial cells (68–70), but it can also occur through squamous epithelial cells in pseudo stratified epithelium (8, 52, 71). During this process, viral particles bind to the epithelial cell surface molecules like heparan sulfate proteoglycans (HSPGs) or Galcer to be transported into the intracellular compartment (52, 71, 81, 82). This process has been observed in oral, intestinal, vaginal and endometrial epithelial cells (52, 83). Viral particles retain their infectious capacity following their translocation into the intracellular space and are released to the external basal space where they can infect intraepithelial lymphocytes or be picked up and transferred by DC or LC. However, the efficiency of transcytosis was found to be as low as 0.01% of the initial viral inoculum (71), and thus, it is highly inefficient.

Furthermore, in one study, most of the translocated viral particles were shown to be retained at the intracellular space in vesicular compartments such as vacuoles or multi-vesicular bodies (MVB) for several days, as observed in tonsil, cervical, and foreskin epithelial cells (8). In these compartments, the virions preserve their infectious capacity and can be the source of infection of CD4^+^ T lymphocytes (84). These retained virions can be released by an inducible exocytic pathway upon stimulation that triggers the reorganization of cortical actin-cytoskeleton, resulting in the release of the viral particles allowing them to infect CD4^+^ target cells at the lamina propria. Such stimulus can be the exposure to pro-inflammatory cytokines (e.g., TNF-α and IFN-γ) or the interaction of epithelial cells with lymphocytes, DC/LC or macrophages, mediated by the adhesion molecules ICAM-1 and LFA-1 (84). Nonetheless, in oral epithelial cells, endocytosis of virions occurs simultaneously with a co-internalization of HSPGs and β-defensins leading to reduced viral infectivity (81). It remains to be determined if a similar process occurs in other epithelia.

Remarkably, viral transmission via transcytosis seems to be more efficient between infected and epithelial cells vs. free viral particles and epithelial cells, likely by the virological synapse formed between the HIV-infected CD4^+^ T cells and the epithelial cells (85).

At this point, some stimuli such as the interaction with viral proteins, and the presence of pro-inflammatory cytokines can trigger both the formation of paracellular passage and the transcytosis of viral particles. Therefore, viral transmigration could be induced *in vivo* by a combination of these mechanisms of viral entry.

Finally, viral entry into the submucosal space could also result from the direct infection of mucosal epithelial cells (86). However, viral permissiveness of these cells is not yet defined, due to the presence of potential restriction factors like TRIM, APOBEC or Tetherin in these cells (47, 48, 71). In fact, a non-productive infection has been reported in oral epithelial cells (47, 48), and also a lack of susceptibility of genital epithelial cells has been described (49). Further investigation related to this topic is needed.

### Trojan Horses and Viral Transfer to Target Cells

#### DC, the Trojan Horses For HIV Infection

**Figure 1:**
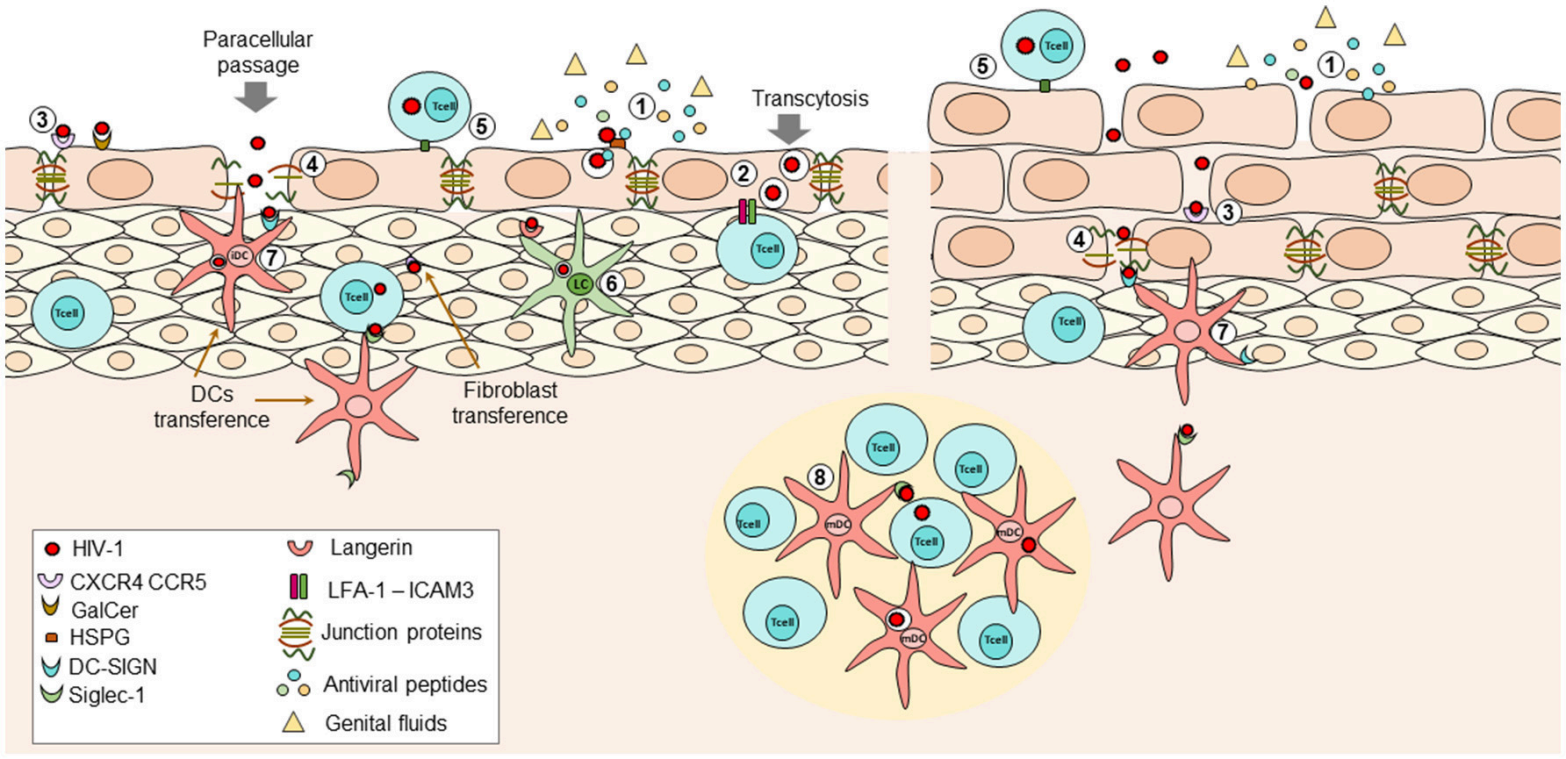
During HIV transmission at female Reproductive Tract (FRT), the initial interactions that occur between the virus and the immune response determine whether the virus is eradicated or if the infection is established and disseminated. (1) At mucosal epithelium presence of genital fluids and antiviral factors such as Beta-defensins, cathelicidin, SLPI, and others, with potent inhibitory capacity against HIV can reduce the infectivity of viral particles; however, these factors may not be enough to avoid the entry of virions into the submucosal space. Viral particles can penetrate the epithelium through physical abrasions that occur during intercourse or by two different mechanisms in intact epithelium: (2) transcytosis; viral particles can bind extracellular receptors expressed on epithelial cells leading to internalization of virions into multivesicular compartments where they retain their infectiveness. After stimuli inducing reorganization of actin-cytoskeleton (e.g., pro-inflammatory cytokines TNF-a or IFN-y, the interaction of LFA-1 on intraepithelial lymphocytes and ICAM-1 on epithelial cells) the virions can be released to infect other susceptible cells. Further, (3) interaction of viral proteins and surface receptors on epithelial cells can induce activation of MCL-MCLK leads to the destabilization of zonula occludens proteins (ZO-1) with the subsequent internalization of occludin and claudins and thus, loss of tight junctions, allowing (4) paracellular passage. The use of one mechanism or another partially relies on the conformational structure of the exposed epithelium. Infected cells can also interact with epithelial cells or susceptible target cells, favoring cell-to cell transmission of the HIV at mucosa (5). Once at the submucosa, the virus can infect intraepithelial cells, or it can bind Langerhans cells or DCs; the first ones can internalize the virions bound to langerin and degrade them, at least in part by induction of autophagy mediated by intracellular Trim5a (6). The second ones can bind the viral particles through DC-SIGN in immature DCs (7) or Siglec-1 in mature DCs (8) once they downregulated DC-SIGN expression. Then, the viral particles are transferred to CD4+ T cells located at the lymphoid nodes, where the activation state of these cells is also crucial for infection. Other recently described cells transferring viral particles are the fibroblast, which are highly ubiquitous and seems to transfer the virus more efficiently than DCs. All these mechanisms have been described in female and male genital mucosa.

Although CD4^+^ T cells are the main HIV target cells, at the genital epithelium DCs are thought to be one of the first immune cells encountering the virus at the submucosa as they are located within outermost genital epithelial layers compared to the CD4^+^ T cells (87) (Figure 1). Also, DCs normally sample the mucosal surface for incoming pathogens, migrating to secondary lymphoid organs for antigen presentation to T-cells (88). The DCs capture virus, and might act as Trojan horses carrying the virus into the lymph nodes, leading to the establishment of systemic infection (89, 90). This type of DCs-mediated viral dissemination has been observed in male (62, 65, 91, 92), female (93) and rectal mucosa (15).

Two main mechanisms have been described for DC's role in viral dissemination: (i) *cis-infection*: The virus binds to the cell membrane molecules CD4 and CCR5, resulting in productive infection of DCs (94). The lack of Birbeck granules in these cells (94) might be related to their susceptibility of infection, as in LC, HIV-1 captured by langerin is internalized into Birbeck granules and partially degraded (95). Indeed, vaginal DCs were shown to exhibit higher HIV replication when compared to skin LC or blood-derived DC, and to preferentially sustain the replication of R5-tropic instead of X4-tropic HIV strains. This peculiar characteristic of vaginal DC may explain at least in part why the majority of mucosal acquired infections are due to R5-tropic HIV (94). The virions released from these DC may then infect CD4^+^ T cells through the immunological synapsis. Nonetheless, the mechanism proposed to account for most of the *in vivo* infections is (ii) *trans-infection* since monocytes-derived DCs are poorly permissive to HIV infection *in vitro* due to the presence of SAMHD1 (96). Furthermore, a specific subset of DC, CD11c^+^CD14^+^, located at ectocervix, endocervix or endometrium was found to have the exclusive ability to capture the HIV without being infected (14). Being bound to the surface of DC, HIV particles have better chances to infect CD4^+^ T cells, mainly at the lymph nodes.

In trans-infection, HIV particles can be internalized by DC or can be bound to the DC surface, in both cases, the virions maintain their infectious capacity until reaching a susceptible cell (97). In the first case, through cell to cell interaction, the viral particles internalized on DC can be transferred via an exocytic pathway, similar to the process of transcytosis in epithelial cells (97). Mature DC are shown to be more effective in transferring viral particles using this strategy, since the virus is stored at non-conventional, non-lysosomal, endocytic compartments (98, 99).

In contrast, most of the internalized viral particles are shown to be quickly degraded in immature DC (100). Thus, HIV particles bound to the DC surface can be more efficiently transmitted to CD4^+^ T cells, vs. those internalized viral particles (13). HIV was shown to bind to DC surface receptors, like DC-SIGN, and to a lesser extent to the Mannose receptor (MR), DC immunoreceptor (DCIR) or GalCer which are predominantly expressed on immature DC (100, 101) or to Siglec-1 molecule, expressed by mature DC (102). The DC isolated from endocervical, ectocervical or endometrial mucosae, can capture HIV particles without the expression of DC-SIGN, suggesting the involvement of other HIV binding molecules on these DC (14). Siglec-1 seems to be the best candidate since it has been considered to play the major role in trans-infection over the one of DC-SIGN (102). In summary, the maturation state of DC may affect their ability to transfer viral particles, as mature DCs are better transferring virions, compared to immature DC. Mature DC preferentially mediate the trans-infection mechanism while the immature DC may mediate both, cis- and trans-infection (13).

Other important aspects affecting viral transfer by DCs are the presence of dendrites, and the proteins implicated in their formation and development. Actin nucleation and an intact cortical actin cytoskeleton were shown to be required to maintain the association of viral particles with dendrites, and prevent the engulfment of the virus into macropinocytic vesicles (103). Interestingly, silencing the expression of proteins that are involved in the actin enucleation and prolongation processes (e.g., TSPN7, DYNM2, and AP2/3 complex), required for dendrites formation, was associated with a decrease in the viral transfer, and an increase in the endocytosis of viral particles (103). In this case, the reduction of dendrites formation resulted in the appearance of short prolongations known as blebs that favor viral internalization (103). Accordingly, decreases of proteins implicated in the endocytic pathway were associated with higher transfer of viral particles to CD4^+^ T cells (103). Nonetheless, it is important to note that viral encounter by mucosal DC can increase the expression of ligands for CCR5 co-receptor, and the antiviral peptides, SLPI and Elafin, suggesting that a protective role against HIV, mediated by DC, should not be ruled out (14).

#### Transfer of HIV Particles to Target Cells by Langerhans Cells, and Stromal Fibroblasts

Langerhans cells (LCs), although first identified in the epidermis are found in stratified epithelia, such as oral and vaginal mucosal epithelium (104, 105). It has been proposed that like DCs, LCs can also transfer HIV particles to target cells (105); however, contrasting findings showed that instead of viral transfer, binding to surface langerin is related to viral internalization and degradation, mediated, at least in part, by intracellular Trim5α that induces the autophagic degradation of HIV, preventing the infection of LCs (106). Nonetheless, such degradation can be abrogated in the presence of high concentration of virus (95). Furthermore, at least in the male mucosa, enrichment of LC-CD4^+^ T cells complexes induced by chemokines secreted by HIV-exposed epithelial cells, have been reported, that could favor cell recruitment and viral dissemination (42, 43, 107). Further studies on the LC's role in viral transfer are required.

Stromal fibroblasts, abundant at all mucosal sites were recently reported to play a role in transferring the HIV-1 particles in one study (17). Hence, fibroblasts from endometrium, cervix, foreskin, and intestines might enhance the infection of CD4^+^ T cells, among other mechanisms, by a trans-infection process, similar to DCs, without being infected (17). Indeed, stromal fibroblasts seem to be more efficient in transferring viral particles, compared to DCs, at least *in vitro*, and can transfer both, X4 and R5- tropic HIV viruses (17). However, stromal fibroblasts do not express DC-SIGN or Siglec-1; receptors responsible for fibroblasts-mediated viral transfer remains to be sought (17). It was further proposed that stromal fibroblasts enhance HIV-1 transmission, perhaps, with viral transfer during the earlier stages of HIV-1 infection; but, once the inflammatory response is instigated, DCs are responsible for viral dissemination (17) (Figure 1). Nonetheless, more studies related to the role of fibroblasts in HIV-1 transfer are required.

### Susceptibility of CD4^+^ T Cells

Finally, a crucial factor in determining the establishment of HIV infection at mucosa is the availability of susceptible target cells, in particular, the CD4^+^ T cells and highly possible, the memory type CD4^+^ T cells (108). In mucosa, there is a large number of CD4^+^ T cells expressing the surface receptor CCR5, for which transmitted/founder viruses have the tropism; thus, the presence of CD4^+^ T cells in mucosa increases susceptibility to infection (109). Nonetheless, not all CD4^+^ T cells subsets are equally infected at early stages of viral transmission. It was observed in the SIV infection model that during SIV vaginal transmission, the Th17 cells (CD4^+^CCR6+, RORγT^+^) constituted the main targets (110). In support, non-infected female sex workers (FSW) from Kenya, have high numbers of cervical Th17 CD4^+^ T cells, but these Th17 cells are depleted in HIV infected FSW (109).

Furthermore, the susceptibility of HIV target cells may vary depending on their localization at the mucosa. The CD4^+^ T cells from ectocervix exhibit the highest susceptibility to HIV, followed by the CD4^+^ T cells at endocervical and endometrial locations, likely due to their Th17 profile and high expression of CCR5 (111). There is also a higher number of intraepithelial CD4^+^ T cells, and macrophages at the ectocervix and the transformation zone compared to the endocervix or vagina, suggesting that HIV transmission, most likely occur at these locations (4, 53).

In addition, the activation status of susceptible target cells plays a critical role in the establishment of HIV infection. Quiescent CD4^+^ T cells can be infected but viral replication in these cells is inefficient (112), while the activated CD4^+^ T cells are permissive to HIV replication (113), producing a high number of viral particles, leading to viral dissemination. Indeed, the presence of a pro-inflammatory local environment, similar to that observed during sexually transmitted infections (STI), favors the establishment of HIV infection, since inflammation mediate the recruiting and activation of CD4^+^ target cells in FRT (114). In fact, elevated mucosal levels of pro-inflammatory cytokines, such as IL-8, IL-1β, IL-α, and TNF-α were associated with increased risk to HIV acquisition (115).

Moreover, the natural resistance to HIV-acquisition, observed in HESN individuals is reproducibly associated with immune quiescence in mucosa (116), characterized by low expression of the activation markers, CD69, HLA-DR, and CD38 on T cells, reduced expression of genes related to the pro-inflammatory response and low production of cytokines (15, 117–119).

Although some findings in the peripheral circulation of HESNs may differ from those in the mucosa, as an elevated immune activation has been described in peripheral blood (120, 121), those results are probably supporting the higher responsiveness to stimuli that HESNs exhibit when compared to healthy individuals who have not been exposed to HIV (122). In summary, whereas immune activation could be higher in peripheral circulation in some HESNs, reduced activation in mucosa is consistently predictive of relative resistance to HIV infection.

### Role of Microbiota in HIV Mucosal Transmission

The interactions between host immune cells and commensal microbial community maintain the homeostatic immune activation status at the mucosal sites in healthy individuals. As immune activation at mucosa is crucial to HIV transmission, the vaginal microbiota may play an interesting role in susceptibility to viral infection. As mentioned before, the first line of antiviral defense is the epithelium, along with the dense amount of glycoproteins forming the glycocalyx, which contain a high concentration of microorganisms constituting the microbiota. A so-called healthy microbiota is identified as a *Lactobacillus* dominant community, with a low microbial diversity (10). Once the virus encounters the mucosae, the low pH and hydrogen peroxide (H_2_O_2_) (123) generated by vaginal bacteria may inactivate the viral particles (124, 125), similar to antiviral peptides, reducing the number of virions that gain access to the epithelium. However, when the microbiota profile changes (i.e., increasing the ecological diversity of microorganisms), the likelihood of HIV transmission may increase, as it has been suggested for bacterial vaginosis (BV) (126). A decrease of vaginal *Lactobacillus* populations and increases in anaerobic and gram-negative bacteria (125), like *Gardenerella vaginalis, Mycoplasma hominis, Prevotella, Mobiluncus*, and *Atopobium* are often observed in BV (127). Remarkably, similar to the anaerobic-induced risk of HIV acquisition reported in women, a 2-year clinical trial, carried out in uncircumcised men, indicated that those who became infected by HIV had higher levels of penile anaerobes than uncircumcised men who remained HIV negative (128).

The microbial dysbiosis, changes in the composition of the ‘healthy' commensal microbiota has been linked with increased pro-inflammatory cytokines in women (129, 130) as well as men (128) and higher proliferation of mucosal T cells, as well as activation/maturation of DC (131), leading to an increase in the number of susceptible target cells for the virus. In fact, the association between the presence of BV and increased risks in HIV acquisition has been reported by several studies (132, 133). However, the reverse does not hold true. Women without the symptoms of BV, but exhibiting low *Lactobacillus* and high diversity communities of microorganisms may have increased activation of the NF-kB signaling pathway, a high concentration of genital pro-inflammatory cytokines and increased activation of CCR5^+^ CD4^+^ T cells at cervical mucosa (9). These asymptomatic women also had a high risk to acquire the HIV infection (134). However, contrasting findings in some cohorts showed that the ratio of *lactobacillus* and pathogenic bacteria in HESNs did not differ from that of healthy donors or HIV positive women (135, 136), underlying the importance of further studies exploring the role of microbiota in HIV transmission.

### Hormonal Regulation in HIV Female Mucosal Transmission

The immune microenvironment at female mucosa is also affected by hormonal fluctuations occurring throughout the menstrual cycle, providing favorable conditions for pregnancy (10). Estradiol and progesterone hormones regulate epithelial cells, T cells, dendritic cells, and others (53). However, such hormonal changes may also increase the risk of acquiring the HIV infection (137). In macaques, high levels of progesterone were related to higher SIV vaginal transmission, with the increased risk also depending on the phase of the menstrual cycle (138). Indeed, a window of vulnerability during the hormonal cycle that lasts for 7–10 days after ovulation has been proposed (53). Also, during the hormonal cycle, the epithelial thickness may vary, mainly by the action of estradiol; low levels of this hormone, in post-menopausal women, and higher levels of progesterone in macaques are related to thinning of the vaginal epithelium increasing susceptibility to HIV infection (53, 138). Furthermore, mucus content and amount also varies according to the menstrual cycle (139).

Hence, the use of hormonal contraceptives has also been linked to higher susceptibility to HIV, as women using injectable or oral contraceptives have a higher frequency of vaginal or cervical CCR5^+^ CD4^+^ T cells in comparison to women control groups, respectively (140, 141). However, the association between the use of contraceptives and the increases in susceptibility to HIV-infection has yet to be shown in cohort studies and remains a controversial issue (10).

## Conclusion

In the context of genital mucosa exposure to HIV, several factors and interactions impact the rates of transmission, as they play crucial roles in determining whether the infection is eradicated at this portal of entry or established and disseminated. In this regard, exhaustive research has been conducted to identify those factors and interactions with the potential to be targeted by preventive strategies. Consequently, some of these factors have been described and studied in detail, leading to a hypothetical model of HIV-1 mucosal transmission, while others remain to be fully elucidated, with the aim to expand our knowledge regarding HIV-transmission. So far, it has been established that the following factors influence the success of viral transmission, and can be considered as potential steps for the development of future preventive strategies: the presence of antiviral peptides; the hormonal levels and the composition of genital fluids; the interaction between the virus and epithelial cells, leading to the loss of the epithelial integrity; the ability of the virus to perform transcytosis through columnar epithelium to reach the submucosal space; the use of different types of cells to transfer, and spread the infection; the predominant vaginal microbiota, and the activation status of the susceptible target cells (Figure 1).

## Author Contributions

SG contributed with literature search and reading and writing and correcting the manuscript. WA-J contributed with writing and suggestions and corrections. MR contributed reviewing the manuscript and writing. R-CS contributed reviewing and writing manuscript.

### Conflict of Interest Statement

The authors declare that the research was conducted in the absence of any commercial or financial relationships that could be construed as a potential conflict of interest.
